# Cost-Effectiveness of Continuously Diffused Oxygen Therapy Compared with Negative-Pressure Wound Therapy

**DOI:** 10.36469/001c.155760

**Published:** 2026-02-10

**Authors:** Matthew Mercurio, Lawrence A. Lavery, Animesh Agarwal, Alisha Oropallo

**Affiliations:** 1 MGM Applied Economic Consulting, Rohnert Park, California; 2 Orthopedics UT Health San Antonio, San Antonio, Texas; 3 Vascular Surgery Donald and Barbara Zucker School of Medicine, Hofstra/Northwell Health, Hempstead, New York

**Keywords:** diabetic foot ulcer, venous leg ulcer, pressure ulcer, chronic wound, continuous diffusion of oxygen, topical oxygen, negative pressure wound therapy

## Abstract

**Background:**

Continuous diffusion of oxygen (CDO) to wounds has demonstrated better effectiveness in healing wounds than negative-pressure wound therapy (NPWT). However, there is limited evidence regarding the cost-effectiveness of CDO therapy and its comparison with NPWT.

**Objectives:**

The purpose of this analysis is to report on the cost-effectiveness of CDO and NPWT using published clinical results as well as real-world clinical outcomes and cost information. The objectives included analyzing the cost-effectiveness of CDO therapy across multiple wound types and anatomical locations, testing the data for robustness, and comparing the cost-effectiveness using results from controlled clinical studies for CDO and NPWT.

**Methods:**

A prospective patients database using real-world clinical results of 764 patients treated using CDO therapy in a broad range of clinical practices across a wide range of wound types and wound locations was analyzed. The clinical data were combined with real world billing data to draw statistically valid cost comparisons. Using 3 methodologies, the cost savings were demonstrated across 2 healthcare systems.

**Results:**

In the US, the average cost savings of CDO was US $14 238 vs NPWT to heal a wound. Kaplan-Meier analysis showed that CDO use in clinical practice had 79.2% full closure in 112 days compared with NPWT, which has 43.2% full closure in the same timeframe for similar wound sizes and severity.

**Discussion:**

There are two primary reasons for the significant cost savings. First, CDO therapy is much easier to apply and maintain than traditional NPWT, as CDO dressings can easily be changed by patients at home without the assistance of a nurse. The second reason is the substantially higher efficacy of CDO therapy.

**Conclusion:**

CDO is highly efficacious in clinical practice and cost-effective compared with NPWT and other therapies such as moist wound therapy and hyperbaric oxygen.

## INTRODUCTION

Patients with chronic wounds pose a significant and growing challenge for healthcare as populations age and the prevalence of diabetes, obesity, and atherosclerosis continues to increase worldwide. In addition, the failure to heal these wounds is associated with additional burdens of infection, sepsis, amputation, and recurrence complications, as well as death from direct complications of the wounds themselves. In the US, chronic wounds affect around 6.5 million patients.^1^

Chronic ulcers are a growing cause of patient morbidity and contribute significantly to the cost of healthcare in the US. The most common causes of chronic ulcers include venous leg ulcers, pressure ulcers, diabetic foot ulcers, and leg ulcers of arterial insufficiency. It is claimed that in 2022, in the US alone, in excess of US $13.8 billion was spent annually on the treatment of chronic wounds, and the burden is growing rapidly due to increasing healthcare costs, an aging population, and a sharp rise in the incidence of diabetes and obesity worldwide.^1^ The annual wound-care product market in the US is projected to reach US $20.2 billion by 2030.^1^ Because these patients are often seen in a variety of settings or simply fail to access the healthcare system, it is difficult to obtain accurate measurements for the total cost burden of chronic wounds.

Statistical modeling using peer-reviewed data to evaluate comparative effectiveness and quality-adjusted life-years (QALYs) between various therapies is an accepted technique. As an example, a recent cost-effectiveness analysis used similar statistical techniques to evaluate 5 advanced skin substitutes.^2^ Moreover, a recent analysis by Chan and Campbell used statistical modeling to compare moist wound therapy (MWT), negative-pressure wound therapy (NPWT), and hyperbaric oxygen (HBO) to continuous diffusion of oxygen (CDO) using peer-reviewed literature combined with actual Canadian healthcare costs.^3^ They found that CDO both improved the QALYs and resulted in dramatic cost savings, ranging from US $1800 vs MWT to US $14 060 vs HBO per wound.

The results of our statistical evaluation build and expand upon those results by including real-world clinical outcomes, further suggesting that CDO therapy reduces economic healthcare burden with a nontrivial increase in quality-of-life outcomes. While CDO therapy is reimbursed in most provinces in Canada and has a nationwide recommendation as a standard of care,^4^ its reimbursement in the US is limited to the Federal Supply Schedule, several states for Medicaid, and commercial payors. The lack of national reimbursement for CDO limits access to the therapy, the associated cost savings, and improvements in clinical outcomes, despite multiple recently published systematic reviews.^4–11^ The inclusion of CDO therapy for wide variety of wound types by healthcare decision-makers would allow for much broader access to this therapy and result in potentially significant healthcare savings.

CDO therapy continuously diffuses pure, humidified oxygen into an oxygen-compromised wound to significantly accelerate wound healing while maintaining a moist wound-healing environment, maintaining patient mobility, and significantly reducing costs. CDO is essentially MWT with the added benefit of a continuous supply of oxygen directly to the tissue. The CDO system is a wearable, low-flow tissue oxygenation and wound-monitoring system that provides a continuous flow of pure, humidified oxygen to wounds such as venous leg ulcers, diabetic foot ulcers, pressure ulcers, burns, and surgical incisions. The CDO system in this analysis consisted of a handheld electrochemical oxygen concentrator, an oxygen delivery extension set, and a wound-site oxygen delivery cannula integrated into an oxygen diffusion dressing. Through the oxygen diffusion dressing, oxygen was provided directly to the wound, evenly and continuously dispersing oxygen across the wound.

As an integral part of the CDO system, the CDO device uses electrochemical oxygen generator cell technology to continuously generate pure humidified oxygen at adjustable flow rates from 3 to 15 mL/h and delivers the oxygen directly to the wound bed environment within the oxygen diffusion dressing. The oxygen diffusion dressing is an all-in-one dressing for medium-to-high exudating wounds designed to allow even distribution of oxygen over the entire wound. The CDO device incorporates enhanced fuel cell chemistry, utilizing a proton exchange membrane to electrochemically generate the low-flow pure oxygen. In simple terms, the CDO device extracts oxygen from room air, concentrates the oxygen through the proton exchange membrane, and creates an oxygen-rich environment by increasing the available oxygen at the wound site under the dressing. The CDO device monitors the flow of oxygen as well as the pressure at the wound site within the dressing, notifying the user if the flow is compromised or if the wound pressure is excessive. The pressure-monitoring feature is particularly useful for preventing pressure ulcers and aiding in the healing of foot ulcers, especially in patients with diabetes. Excessive or prolonged pressure can damage tissues and compromise blood flow, slowing or preventing wound healing. For diabetic foot ulcers, devices that monitor pressure ensure the effectiveness of “off-loading,” the process of taking pressure off the wound site. The battery-operated device is lightweight, wearable, and can be worn discreetly, functioning in remote communication with the wound site through long microbore tubing to enable monitoring of the wound bed conditions. Unlike intermittent therapies that require the patient to remain in place, a wearable CDO device is lightweight and silent, allowing the patient to be mobile and continue daily activities. The portability makes it suitable for use in a home care setting, reducing the need for frequent hospital check-ups.

Epithelialization is an essential component of wound healing and is used as a defining parameter of successful wound closure. A wound cannot be considered healed in the absence of re-epithelialization, and the epithelialization process is impaired in all types of chronic wounds. For purposes of this analysis, a successful (100%) wound closure is defined as complete re-epithelialization without drainage or dressing requirements.^12^ With regard to the comparative NPWT devices used in this analysis, NPWT was not used to take a wound to full closure, and so generally, moist wound therapy (MWT) was used to finish the treatment (hence, one will sometimes see the combined term NPWT/MWT utilized for therapy involving an NPWT-treated wound) and the term “healed” is used. CDO therapy, however, can take a wound to full closure without additional therapy. We statistically evaluated the costs of CDO and NPWT using real-world costs combined with clinical performance in terms of time to healing and overall efficacy for both CDO and NPWT, for all cases and studies analyzed.

## METHODS

This study analyzed the cost-effectiveness of CDO and NPWT using published results and real-world clinical outcomes and cost information following STROBE guidelines.^13^ We present a statistical evaluation of the cost-effectiveness of CDO therapy across multiple wound types and locations, and the cost-effectiveness compared with NPWT. We have reported the methods and results from the comparative clinical efficacy analysis used for this cost analysis elsewhere.^14^ More detail on the handling of data, testing for robustness, statistical methods, and a discussion regarding the advantages and limitations of the study design and analysis can be found therein. That previous study included a comprehensive methodology section, statistical rigor, and full robustness testing, including proportional hazards validation using Schoenfeld residuals, which demonstrated remarkable consistency across all wound types, anatomical locations, age groups, and sex. Notably, no element failed the proportional hazards assumption, which is rare in clinical datasets and confirms underlying coherence and validity of the results. The clinical data from this previous study were combined with real world billing data to draw statistically valid cost comparisons. The data for CDO therapy used real-world data, which included clinical outcomes and cost data for multiple wound types and anatomical locations, and compares it to the cost-effectiveness using results from controlled clinical studies for CDO and NPWT. Although the majority of the wounds considered in this analysis were diabetic foot ulcers, a significant number of other wound types are also involved in the comparison of the two therapies, including but not limited to pressure ulcers, leg ulcers, burns, and surgical wounds. Thus, this report demonstrates both the cost-effectiveness of CDO in a wide variety of wound types and locations, and the cost-effectiveness of CDO therapy vs NPWT. In this context, cost-effective or cost-effectiveness was defined as when two or more activities provided the same or a similar level of benefits and the least costly activity providing that level of benefits was unambiguously preferred.

The availability of cost data for both CDO and NPWT allowed us to combine reported clinical effectiveness data with basic diagnosis data to make statistically valid cost comparisons between the two therapies. Various statistical models are used to measure the cost for a given level of efficacy, or alternatively the level of efficacy for a particular cost of both CDO and NPWT.

### Data Sources

For the real-world clinical results using CDO therapy, a prospective patients database (PPD) of 1181 patients treated using CDO therapy using the OxyGeni® (formerly TransCu O2®) device was used (EO2 Concepts). Clinical data was collected for patients in the PPD who were treated with CDO therapy during regular clinical practice. Patients voluntarily consented to the collection. This PPD was first published online on May 10, 2011, and was designed to capture real-world experience with CDO. Inclusion criteria were broad, since this was intended to be a real-world analysis of actual clinical use. Inclusion criteria included age older than 18 years, written consent to have clinical data gathered, no enrollment in a controlled clinical trial for wound care, and compliance with CDO therapy treatment guidelines. In addition, exclusion criteria included any documented evidence of noncompliance with CDO therapy in the clinical records. Records were kept in a secure, restricted-access location following HIPAA standards. De-identified records were made available for data analysis.

**Figure 1.**
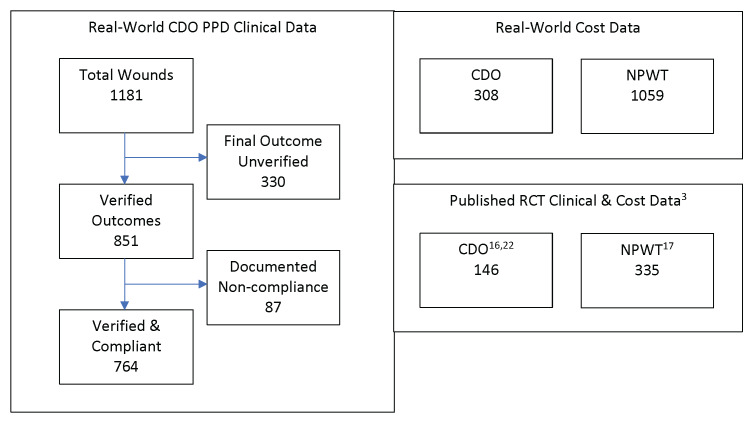
Datasets Used in Cost-Effectiveness Analysis Abbreviations: CDO, continuous diffusion of oxygen; NPWT, negative-pressure wound therapy; PPD, prospective patients database.

The PPD records contain baseline data and routine follow-up records from clinicians’ notes for patients treated with CDO at a variety of clinics. The PPD was designed to capture basic baseline wound data such as wound size, type, and age, as well as patient age and sex. Other baseline data that can affect wound healing, such as infection status, vascular status, comorbidities, HbA1c level, off-loading, and nutritional status, were not routinely tracked or reported by the clinics and are therefore not considered here. The overwhelming majority of the records are from wound care clinics. The data entries from those records in the PPD were verified by a third-party clinician. Patients whose records could not be verified were excluded from this analysis of the PPD. Excluded from this analysis are 330 records that do not list a final verified outcome (ie, healed vs not healed). Most of the excluded records were from patients who were lost to follow-up or for whom a final treatment record could not be obtained. While this number appears high compared with controlled studies, it is more reflective of real-world scenarios, where patients do not return for a variety of reasons.^15^ This leaves 851 valid patient records upon which this analysis is based. These statistics are based on whatever data are available for each measure: some records had data points that were missing or could not be verified for a particular outcome. Patients for whom no information on the outcome of the therapy (Treatment Outcome = Clinical Data Insufficient) were excluded from the efficacy calculations entirely. Thus, Success = Healed/(Healed + Goals Not Met). The patients in the PPD were seen and treated over an 8-year period, September 2009 through May 2017, and, as such, the patients entered the analysis database continuously throughout this time period. Of these 851 patients, 87 had documented noncompliance in the clinical records. These patients were also excluded, resulting in a total of 764 patients who met all inclusion and exclusion criteria. **Figure 1** depicts the various datasets used in this analysis, including published clinical trial data for comparison with the PPD and real-world cost data.

Two datasets were used for the comparison with clinical data published in controlled clinical trials for diabetic foot ulcers, one each for CDO and NPWT. For CDO, the study of 146 patients in a placebo-controlled trial led by Armstrong utilizing the TransCu O2® device (EO2 Concepts) was used.^16^ This study was used since the design was comparable to the NPWT study, and it was referenced in multiple systematic reviews and meta-analyses.^5–11^ For NPWT, the study of 335 patients in a trial using a variety of MWT therapies as the control by Blume et al,^17^ utilizing the V.A.C.® device (3M/KCI), was used. The Blume et al study was used as a comparator for multiple reasons. First, the 2 clinical CDO datasets described above were initiated shortly after the publication of the Blume et al study in 2008. The PPD was initiated approximately 1 year afterward in fall 2009. The design of the CDO placebo study was designed in consultation with the Centers for Medicare & Medicaid Services (CMS) starting in 2011 and used the Blume et al study as a comparator since it addressed the same wound type, similar criteria, and was considered the leading NPWT publication at the time. The risk ratio of 1.48 reported by Blume et al is comparable to the results reported in recent systematic reviews and meta-analyses, further justifying its use as a realistic, if not conservative, comparison for this analysis. Kadavan et al^18^ cited it “as influential based on studentized residuals,” indicating that it had a large, positive impact on the final, combined results of other analyzed studies. In other words, Blume et al was found to be a statistical outlier that pulled the overall meta-analysis result toward its own findings. While the Kadavan et al meta-analysis focused on odds ratios and did not report risk ratios, those in earlier meta-analyses did. The overall risk ratios reported in the reviews by Liu et al^19^ and Huang et al^20^ are similar or lower (1.48 and 1.41, respectively) than that reported by Blume et al, showing not only a very consistent performance over time in multiple studies, yet also confirming the findings of Kadavan et al and the use of the Blume et al study as a comparator in the current analysis. Finally, CMS has referred to the Armstrong CDO placebo study as the “gold standard” for study design. Parameters from Blume et al were considered in consultation with CMS during the design of the CDO placebo study. These two datasets and the PPD dataset were analyzed using comparative effectiveness research (CER) tools.

These clinical data were paired with cost-of-treatment data using billing records on patients during the timeframe November 2020 through December 2021 from Homelink, the nation’s largest ancillary provider group. Because the PPD and clinical trial data do not include cost data, data on the costs of CDO treatment are derived from billing data provided by Homelink and include (1) the cost per day for rental of the CDO equipment, (2) the cost of disposable wound care supplies, and (3) the costs of weekly physician or nursing visits for wound debridement.

Although the Homelink data are not the actual cost data associated with the PPD data (CDO treatment) or the Blume et al study (NPWT), they are real-world data from the actual application of both therapies, and there are distinct advantages to utilizing the Homelink cost data for both CDO and NPWT:

The CDO cost database provided by Homelink included 308 patients treated with CDO therapy and includes the number of “days on service” (ie, the amount of time the patient was treated using CDO therapy) and the total cost of CDO therapy, from which the daily cost can be derived.

The NPWT cost database provided by Homelink included 1059 patients treated with NPWT and the rental duration and cost for the vacuum equipment, the cost of disposable supplies used, and the nursing costs associated with changing the dressings for NPWT. Thus, the Homelink databases provided a methodology for direct comparison between the costs of CDO vs NPWT.

Both the PPD as well as the Homelink CDO and the Homelink NPWT databases have been augmented with either *International Classification of Diseases, Ninth Revision* or *Tenth Revision* (ICD-9 or ICD-10) diagnosis data. This augmentation facilitates the ability to compute the costs for the CDO patients in the PPD as if they had been treated with NPWT based on their days on CDO therapy.

### Comparative Effectiveness Research

The effect of interventions designed to help prevent or treat significant wounds can be assessed through different study designs, including both retrospective and prospective cohort studies. In contrast to strictly one or the other of these two paradigms, this study is a retrospective comparative study using prospective data collection. In both the US and Europe, there was an increased interest in comparative (or relative) effectiveness of interventions to inform health policy decisions. In the US, the American Reinvestment and Recovery Act established a federal coordinating council for comparative effectiveness research (CER). This council defined CER as the “conduct and synthesis of research comparing the benefits and harms of different interventions and strategies to prevent, diagnose, treat and monitor health conditions in ‘real world’ settings.”^21^ It noted that the purpose of this research was to inform patients, providers, and decision-makers, responding to their expressed needs, about which interventions are most effective for patients under specific circumstances. To provide this information, CER must assess a comprehensive array of health-related outcomes for diverse patient populations. Interventions may include medications, procedures, medical and assistive devices and technologies, behavioral change strategies, and delivery system interventions. Furthermore, it noted that CER necessitates the development, expansion, and use of a variety of data sources and methods to assess comparative effectiveness.

### Cost of Therapy

To calculate the total cost of CDO therapy for all patients in the PPD, an attempt was made to match each PPD patient with the average cost figures from the Homelink cost data using the full 6-digit ICD identifier. In cases where no such match could be made, a further attempt was made to match each patient in the PPD to the 3-level ICD (ie, the first 3 digits of the ICD-9/ICD-10 indicator). Finally, in the cases where even that match could not be made, costs were assigned to each PPD patient based on the average of costs across all Homelink patients, including all ICD-9/ICD-10 figures.

There were 3 ways to develop the costs for patients treated with NPWT to compare with CDO costs, given that we did not have an individual listing of NPWT patients (as we did in the PPD for CDO patients). The first methodology was to rely on prior studies that compute the costs for both therapies. The second methodology used Homelink cost data to compare the costs of CDO from the PPD and NPWT using the Blume et al study. The third methodology assessed the costs for the patients treated with CDO therapy as though they had been treated with NPWT, again applying the costs from the Homelink database.

The first method was previously performed and published by Chan and Campbell, who computed the cost of both CDO and NPWT based on actual Canadian health system costs, reported in US dollars, using outcomes from published studies for CDO and NPWT.^3^

The second methodology for comparing both the efficacy and the costs of CDO and NPWT was to use the Homelink data to assess costs in the Blume et al study. An attempt first was made to match each PPD patient with the average cost figures from the Homelink cost data using the full 6-digit ICD identifier. In cases where no such match could be made, a further attempt was made to match each patient in the PPD to the 3-level ICD (ie, the first 3 digits of the ICD-9/ICD-10 indicator). Finally, in the cases where even that match could not be made, costs were assigned to each PPD patient based on the average of costs across all Homelink patients, including all ICD-9/ICD-10 figures.

The third methodology for comparing the costs between CDO and NPWT was to assess the costs for the patients treated with CDO therapy as though they had been treated with NPWT, again applying the costs from the Homelink database. For this comparison, we again relied on 1 line for each patient, and all 3 datasets include the field ICD-9 or ICD-10 for each patient. Thus, we can merge the Homelink data for NPWT against the PPD for CDO patients using ICD-9/ICD-10, and then recompute the costs as if each CDO patient was in fact treated with NPWT. As with the attempt to match the CDO efficacy data with the CDO cost/billing data (described above), in the first round, an attempt was made to match the NPWT cost data to the PPD at the full ICD-9/ICD-10 level. For those patients who did not match at this level, we then attempted to match at the top-level of ICD-9/ICD-10 (ie, the first 3 digits before the secondary ICD field). Any remaining nonmatches were assigned a cost equal to the weighted overall cost over all patients in the Homelink NPWT cost/billing data.

## RESULTS

### Efficacy of CDO Therapy from PPD

The high efficacy of CDO therapy from the PPD across a wide range of wound types has been reported previously^14^ and is summarized briefly here since it is used to calculate the overall cost of therapy to treat wounds. In addition to diabetic foot ulcers, venous leg ulcers, pressure ulcers, and surgical wounds, other wound types that have been successfully treated with CDO include radiation burns; arterial and mixed-etiology leg ulcers; dehisced wounds (face, breast, chest, abdomen, leg, foot, and toe); toe, partial foot and leg amputations; skin and tissue grafts; frostbite; tunneling wounds; traumatic wounds; and plastic surgery (facelift, breast reduction, nipple salvage, and cobalt laser facial ablation). **Table 1** summarizes the results from the PPD by wound type. Although many of the other measures in **Table 1** were based on patients treated and tracked in the PPD, the success outcomes were only for patients who could be verified and were compliant with the therapy regimen (764 patients), so that the outcomes could be compared with controlled studies where compliance is a requirement. This corresponds to an overall, real-world compliance rate of 89.8%. Further details regarding the clinical efficacy of CDO in the PPD are available in the paper by Mercurio et al.^14^

**Table 1.** Summary of Results from Continuous Diffusion of Oxygen Using Prospective Patients Database

**Parameter^a^**	**Total, All Wounds**	**Diabetic Ulcers**	**Venous Ulcers**	**Pressure Ulcers**	**Surgical Wounds**	**All Other**
No. of patients treated	764	200	111	145	145	163
Success rate, overall, %	76.3	75.5	71.2	71.7	84.1	77.9
Success rate, Medicare age, %	72.7	73.2	67.0	73.4	77.1	73.3
Patient age, y (mean ± SD)	66.1 ± 14.7	65.5 ± 13.2	70.8 ± 14.9	68.8 ± 16.5	61.2 ± 14.6	65.3 ± 13.1
Medicare age (≥65 y), %	49.1	50.5	65.7	54.5	38.6	38.8
Medicare age, y (mean ± SD)	78.0 ± 8.8	75.8 ± 8.4	79.8 ± 8.5	81.1 ± 8.4	75.0 ± 8.7	78.4 ± 8.7
Wound age, days (mean)	298.3	292.4	440.3	332.4	164.2	292.0
Wound size, cm^2^ (mean ± SD)	11.7 ± 28.6	6.1 ± 9.0	20.5 ± 34.4	10.5 ± 20.4	7.1 ± 14.6	17.5 ± 43.9
Max. patient age healed, y	101	97	101	100	100	93
Max. wound size healed, cm^2^	283	56	224	85	122	283
CDO therapy days, mean	65.0	66.0	85.7	67.6	52.8	58.2

Abbreviations: CDO, continuous diffusion of oxygen; Max., maximum.

^a^Parameters such as patient age, wound age, and wound size are baseline prior to application of CDO. Wound age indicates how long the wound was noted to be open prior to application of CDO. CDO therapy days indicate how long CDO therapy was applied, regardless of outcome. Medicare age refers to the portion of patients aged 65 years or older at the start of CDO therapy.

### Real-World Cost and Effectiveness Comparison: CDO vs NPWT

**Figure 2.**
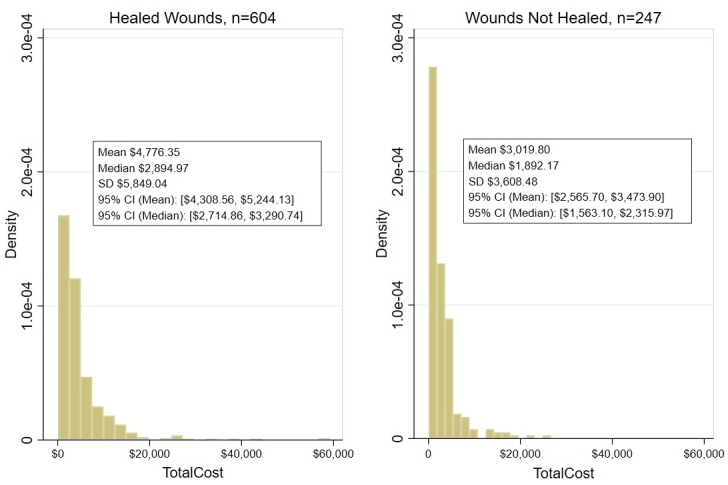
Estimated Total Cost of Wound Treatment (US $) for Continuous Diffusion of Oxygen Using Prospective Patient Database Abbreviation: CI, confidence interval.

The total cost to treat a wound using CDO therapy for all patients in the PPD is shown in **Figure 2**. These costs include all wound types and locations. **Figure 2** breaks out the costs of CDO therapy separately for healed wounds vs non-healed wounds.

To compare the cost of treatment between CDO and NPWT, we first compared the primary summary statistics for clinical efficacy between the two therapies, summarized in **Table S1**.

The proportion of foot ulcers that achieved complete ulcer closure with NPWT was 43.2% (73/169) within the 112-day active treatment phase. Evaluating the same statistic in the PPD, we found that after 112 days of therapy, the proportion of ulcers that achieved complete ulcer closure with CDO was 79.2%. Thus, almost twice as many patients had their wounds healed within 112 days using CDO therapy as with NPWT.

The Kaplan-Meier median estimate for 100% ulcer closure with NPWT was 96 days (95% CI: 75.0, 114.0). If we evaluate the same statistic in the PPD, we find that the median estimate for 100% ulcer closure was 58 days (95% CI: 54, 72). Again, wounds healed 66% faster using CDO compared with NPWT.

The first method of cost comparison was to use prior studies that compute the costs for both therapies. This had been performed and published by Chan and Campbell, who computed the cost of both CDO and NPWT based on actual Canadian health system costs, reported in US dollars, using outcomes from published studies for both CDO and NPWT.^3^ The clinical outcomes are shown in **Table S2**. The authors reported a significantly lower overall per patient cost of CDO vs NPWT, or cost savings, ranging between US $4940 and US $6455, shown in **Table S3**. The cost differential was calculated as: Cost of NPWT - Cost of CDO. In other words, the cost differential was the cost savings achieved by using CDO therapy instead of NPWT. The cost differential increased as wounds became larger, were more chronic, or were on weight-bearing (plantar) surfaces.

**Figure 3.**
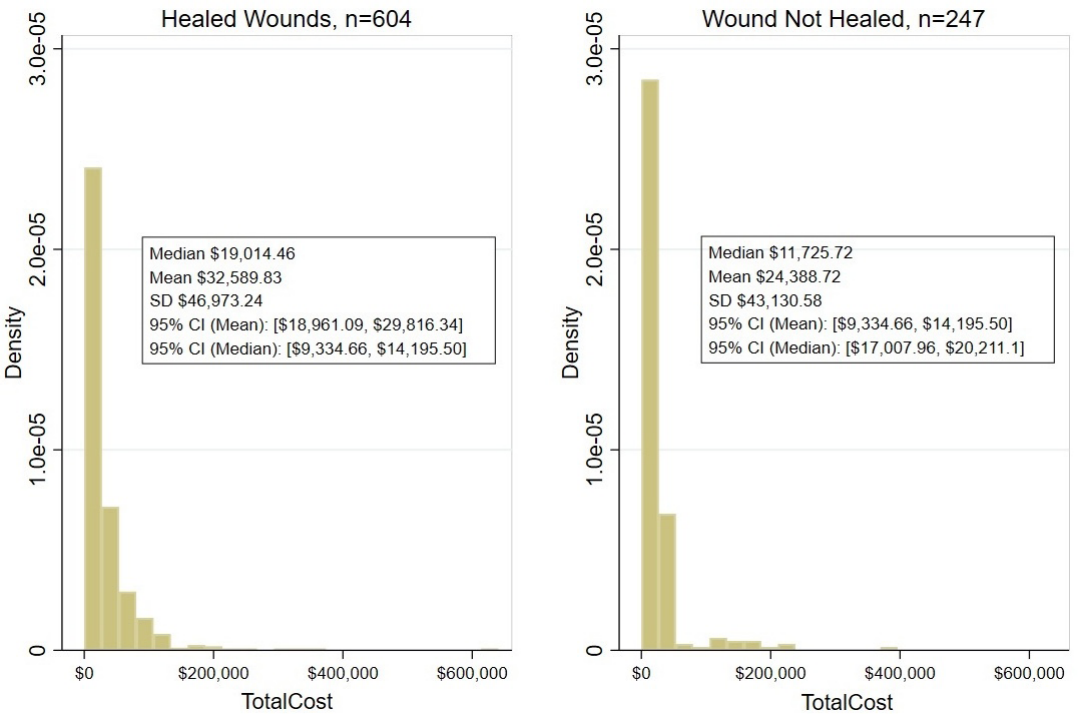
Estimated Total Cost of Wound Treatment (US $) for NPWT Using Blume et al Study Clinical Outcomes Abbreviations: CI, confidence interval; NPWT, negative-pressure wound therapy.

The second methodology for comparing the costs of CDO and NPWT used the Homelink data to assess costs in the Blume et al study. **Figure 3** demonstrates the costs of NPWT based on the patient outcomes in Blume et al combined with cost data for NPWT patients in the Homelink billing data.

**Figure 4.**
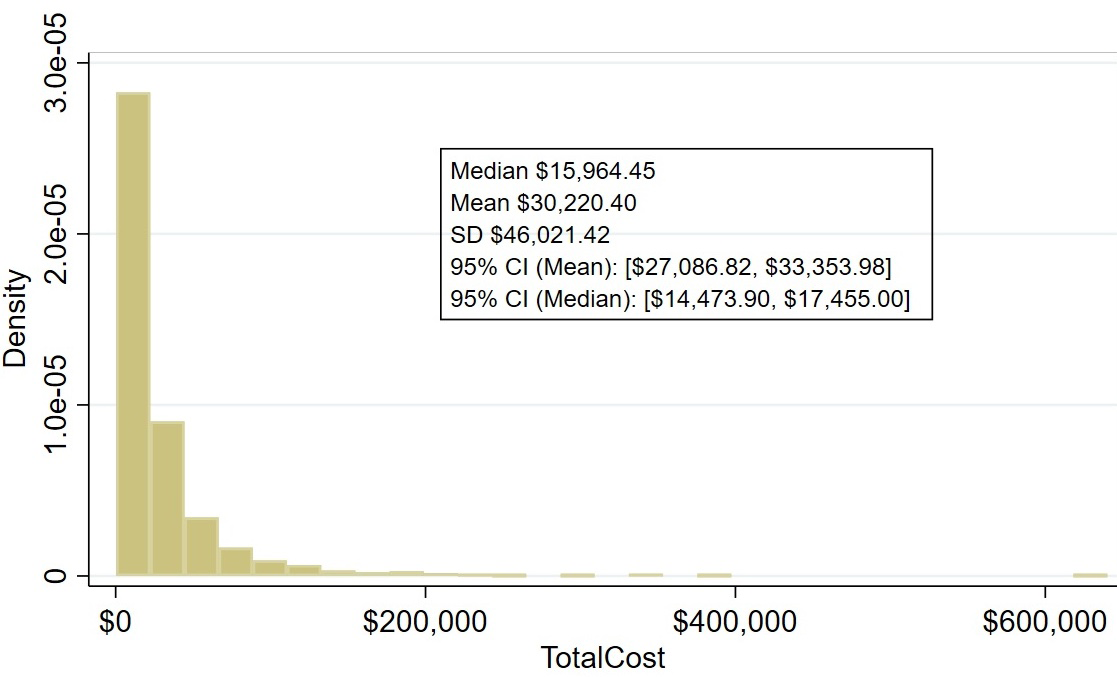
Estimated Total Cost of Wound Treatment (US $) for NPWT Assuming CDO PPD Patients Had Been Treated with NPWT Abbreviations: CI, confidence interval; CDO, continuous diffusion of oxygen; NPWT, negative-pressure wound therapy; PPD, prospective patient database.

The third methodology for comparing the costs between CDO and NPWT assessed the costs for the patients treated with CDO therapy as though they had been treated with NPWT, again applying the costs from the Homelink database. **Figure 4** demonstrates the costs of therapy, assuming the patients treated with CDO had instead been treated with NPWT.

By reference to any of these three cost estimation methods, the fundamental cost differences in the US between CDO and NPWT were extreme, as shown in **Table 2** for methodologies 2 and 3, with the cost of CDO therapy being approximately 3 times lower on average and almost a full order of magnitude less expensive measured by median than NPWT.

A *t*-test of any variety, with any assumption regarding the equality/nonequality of the variances between the two patient samples, demonstrated statistically significant differences in cost at far beyond the 99% confidence level, for all patients, healed wounds only, or any other possible subset of the two populations.

**Table 2.** US Cost Comparison to Treat Wound Using Continuous Diffusion of Oxygen and Negative-Pressure Wound Therapy

	**CDO, US $**	**NPWT, US $**	**Difference, US $**
All wounds			
Mean	4268.86	15 964.45	11 695.59
Median	2694.08	30 220.40	27 526.32
SD	5357.35	46 021.42	
Healed wounds			
Mean	4776.35	19 014.46	14 238.11
Median	2894.97	32 589.83	29 694.86
SD	5849.04	46 973.24	
Non-healed wounds			
Mean	3019.80	11 725.72	8705.92
Median	1892.17	24 388.72	22 496.55
SD	3608.48	43 130.58	

Abbreviations: CDO, continuous diffusion of oxygen; NPWT, negative-pressure wound therapy.

## DISCUSSION

### Comparison of CDO to NPWT

All three different methods used to calculate and compare the costs of CDO and NPWT resulted in statistically significant cost savings of CDO over NPWT. Moreover, CDO has been shown to have significantly lower costs in two different healthcare systems: Canada and the US. For the Canadian cost differential using the first methodology, CDO has been shown to have an average per-wound savings of US $4940 vs NPWT. These cost savings increased as wounds become more difficult to heal (larger or more chronic wounds). This is because the relative efficacy of CDO has been shown to increase as wounds become more chronic.^3,16,22^ CDO has also been shown to have per wound costs savings vs moist wound therapy (US $1860) and hyperbaric oxygen therapy (US $14 060) in Canada.^3^ The analysis of costs in the US using the second and third methodologies yielded an average cost savings per wound treated of US $11 696 overall (including nonhealed wounds) and US $14 238 to bring wounds to full closure (wounds that healed).

There are two primary reasons for the enormous cost differences between CDO and NPWT. On the purely cost end, the NPWT cases reviewed here relied on a nurse, nurse practitioner, or other professional for help in routine wound cleaning and dressing, whereas CDO does not require such costly intervention. CDO dressings can easily be changed by patients at home without the assistance of a nurse. Since the CDO dressings are simple to apply, NPWT will always have significantly larger daily costs (all else held equal). For example, in the CDO diabetic foot ulcer study analyzed, 145 of the 146 patients were able to change dressings themselves or with the assistance of a companion.^16^ The second reason for the higher cost of NPWT is that, because of the substantially lower efficacy in wound closure, NPWT takes longer than CDO therapy to close wounds, sometimes substantially longer because of the lower efficacy of NPWT.

Because individual-level clinical and cost data for NPWT were not jointly observed, traditional regression-based adjustment techniques such as propensity score matching were not applicable without introducing unsupported assumptions. Instead, robustness was assessed through multiple independent cost-comparison methodologies, all of which produced consistent and statistically significant results.

Instead, the analytical approach employed here focused on estimating costs associated with observed levels of clinical effectiveness, rather than attempting to model treatment assignment mechanisms. This approach was consistent with established health-economic and comparative effectiveness research methodologies when real-world data are paired with published trial results. Robustness of the economic findings was evaluated using three independent costing methodologies, including (1) previously published system-level cost analyses, (2) real-world billing data applied to published NPWT outcomes, and (3) counterfactual costing of CDO-treated patients as if they had been treated with NPWT using matched diagnostic codes. The strong convergence of results across these distinct approaches provides assurance that the observed cost differentials were not driven by any single cost assumption or modeling choice.

In our previous publication describing clinical efficacy of CDO and NPWT in greater detail, Kaplan-Meier estimates were used to compare the clinical efficacy of CDO to NPWT, since they are most often used to report results for NPWT.^14^ As shown in **Table S1**, which compares the real-world results for CDO to the clinical trial results for NPWT, full closure is achieved 66% faster using CDO. Similar results are found in the data for full closure achieved at 112 days: CDO closed 83% more wounds relative to NPWT. The wound sizes are similar between the two treatments, with CDO averaging 11.7 cm^2^ and NPWT averaging 13.5 cm^2^. The wounds in the CDO PPD ranged from stage 1 ulcers to exposed bone and cartilage, including dehisced wounds and wounds that failed treatment with standard of care. Kaplan-Meier analysis, combined with proportional hazard test and analysis, showed that CDO therapy is not only efficacious, but the data is also robust across sexes, wound types, and anatomical locations. Clinical efficacy was equivalent regardless of age and sex and across all wound types studied, which includes wounds ranging from chronic ulcers of all types to acute burns and surgical incisions, as well as various anatomical locations ranging from head to toe. The Medicare-aged population has similar efficacy to the general population. The validity of this comparison was supported by the similarities in the datasets, robustness of the CDO PPD data, and similarity of the CDO PPD results to published clinical trial results.

From the two clinical trial publications of both treated diabetic foot ulcers shown in **Table S2**, similar trends can be observed. NPWT closed fewer wounds (43% vs 46% for CDO) and yet required longer to do so (16 weeks for NPWT vs 12 weeks for CDO). This difference becomes larger when one considers that approximately 9% of wound closures included in the NPWT results were achieved through surgical closure, grafts, or other methods, reducing the efficacy to approximately 39% without other intervention. It is generally known that NPWT does not take wounds to full closure by itself. In the CDO study, only CDO was used to take wounds to full closure. If the clinical outcomes are adjusted to remove other interventions from the NPWT results, the clinical efficacy differences shown here become even more significant. Given the similarities in the datasets for the clinical trials and PPD, the robustness of the CDO PPD data, and the similarity of the CDO PPD results to published clinical trial results, the higher performance of CDO compared with NPWT is a valid comparison.

This analysis should be interpreted within the context of a comparative effectiveness and cost-effectiveness framework rather than as formal causal inference. The study did not rely on randomized treatment assignment and therefore cannot claim causal superiority in the strict statistical sense. Rather, it evaluated comparative clinical performance and associated costs under real-world conditions using a combination of prospectively collected observational data, published randomized trial outcomes, and real-world billing records.

Accordingly, these conclusions emphasize comparative performance and economic efficiency rather than causal attribution. The observed differences in healing rates, time to closure, and costs reflect reproducible patterns across independent data sources and analytic methods, and they provide clinically and economically meaningful information for decision-makers operating in real-world care environments. These results complement, rather than replace, evidence from randomized controlled trials and should be viewed as part of a broader evidence base informing wound-care policy and practice.

The evidence provided here supports and supplements not only the high efficacy of CDO therapy compared with other wound therapies but also the significant cost savings that can be achieved through broad implementation of CDO therapy. Based on recent publications of robustly designed studies, CDO therapy has numerous recommendations as a standard of care from organizations such as the American Diabetes Association, the Wound Healing Society, Health Canada, and the International Working Group for the Diabetic Foot, among others. The inclusion of CDO therapy as an option for the treatment of a wide variety of wound types by additional healthcare decision-makers would allow for much broader access to this therapy and could result in potentially significant healthcare savings.

## CONCLUSION

Using real-world costs, CDO demonstrated significant cost savings compared with NPWT in both the US and Canadian healthcare systems. These savings are driven by ease of application and higher efficacy. Adoption of CDO could reduce healthcare costs while improving clinical outcomes.

## Supplementary Material

Online Supplementary Material
