# Supplementary material for: Cost-Effectiveness of Continuously Diffused Oxygen Therapy Compared with Negative-Pressure Wound Therapy
Source: J Health Econ Outcomes Res. 2026 Feb 10;13(1):30–8. doi: 10.36469/001c.155760 (PMC12900533; doi:10.36469/001c.155760)
Supplement: Online Supplementary Material [file jheor_2026_13_1_155760_330144.pdf]

Journal of Health Economics and Outcomes Research

Online Supplementary Material

Cost-Effectiveness of Continuously Diffused Oxygen Therapy Compared with Negative-Pressure Wound Therapy. *JHEOR*. 2026;13(1):30-38. doi:10.36469/jheor.2026.155760

Table S1: Comparative Clinical Efficacy of CDO and NPWT: Prospective Patient Database to Blume

Table S2: Comparison of Efficacy of CDO Therapy vs NPWT in Published Studies

Table S3: Canadian Cost Savings of CDO Therapy vs NPWT

This supplementary material has been provided by the authors to give readers additional information about their work.

This is an open-access article distributed under the terms of the Creative Commons Attribution 4.0 International License (CCBY-4.0). View this license's legal deed at http://creativecommons.org/licenses/by/4.0 and legal code at http://creativecommons.org/licenses/by/4.0/legalcode for more information.

Table S1. Comparative Clinical Efficacy of CDO and NPWT: Prospective Patient Database to Blume

Outcome	CDO (Real-World, PPD)	NPWT (Blume Study)
Closure at 112 days, %	79.2	43.2
Kaplan-Meier estimate to full closure, days	58	96
Average baseline wound size, cm^2 (SD)	11.7 (27.7)	13.5 (18.2)

Abbreviations: CDO, continuous diffusion of oxygen; NPWT, negative-pressure wound therapy PPD, prospective patient database.

Table S2. Comparison of Efficacy of CDO Therapy vs NPWT in Published Studies

Study	*N*	Study Length, Weeks	Control Arm	Wound Closure, Study Group	Wound Closure, Control Group	*P*	Relative Risk
CDO[16]	146	12	Placebo system	46%	22%	0.016	2.04
NPWT[17]	335	16	MWT (various)	43%	29%	0.007	1.48

Abbreviations: CDO, continuous diffusion of oxygen; MWT, moist wound therapy; NPWT, negative-pressure wound therapy PPD, prospective patient database.

Table S3. Canadian Cost Savings of CDO Therapy vs NPWT

Wound Type	Overall	In Larger Wounds	With Frequent Debridement	In Chronic Wounds
Cost differential, US $[a]	4940	5450	6100	6455

Abbreviations: CDO, continuous diffusion of oxygen; NPWT, negative-pressure wound therapy PPD, prospective patient database.
[a]Cost differential dollar value represents NPWT Cost – CDO Cost in US $ (cost savings of using CDO vs NPWT).
